# Clinical Characteristics of Mitochondrial Encephalomyopathy, Lactic Acidosis, and Stroke-Like Episodes

**DOI:** 10.3390/life11111111

**Published:** 2021-10-20

**Authors:** Hueng-Chuen Fan, Hsiu-Fen Lee, Chen-Tang Yue, Ching-Shiang Chi

**Affiliations:** 1 Department of Pediatrics, Tungs’ Taichung Metroharbor Hospital, Wuchi, Taichung 435, Taiwan; t11578@ms.sltung.com.tw (H.-C.F.); yu141315@gmail.com (C.-T.Y.); 2 Department of Medical Research, Tungs’ Taichung Metroharbor Hospital, Wuchi, Taichung 435, Taiwan; 3 Department of Rehabilitation, Jen-Teh Junior College of Medicine, Nursing and Management, Miaoli 356, Taiwan; 4 Department of Life Sciences, Agricultural Biotechnology Center, National Chung Hsing University, Taichung 402, Taiwan; 5 Department of Pediatrics, Taichung Veterans General Hospital, Taichung 407, Taiwan; leehf@hotmail.com.tw

**Keywords:** MELAS, mitochondrial DNA, genetics

## Abstract

Mitochondrial encephalomyopathy, lactic acidosis, and stroke-like episodes (MELAS) syndrome, a maternally inherited mitochondrial disorder, is characterized by its genetic, biochemical and clinical complexity. The most common mutation associated with MELAS syndrome is the mtDNA A3243G mutation in the MT-TL1 gene encoding the mitochondrial tRNA-leu(UUR), which results in impaired mitochondrial translation and protein synthesis involving the mitochondrial electron transport chain complex subunits, leading to impaired mitochondrial energy production. Angiopathy, either alone or in combination with nitric oxide (NO) deficiency, further contributes to multi-organ involvement in MELAS syndrome. Management for MELAS syndrome is amostly symptomatic multidisciplinary approach. In this article, we review the clinical presentations, pathogenic mechanisms and options for management of MELAS syndrome.

## 1. Introduction of Mitochondria

### 1.1. Structure of Mitochondria

**Figure 1:**
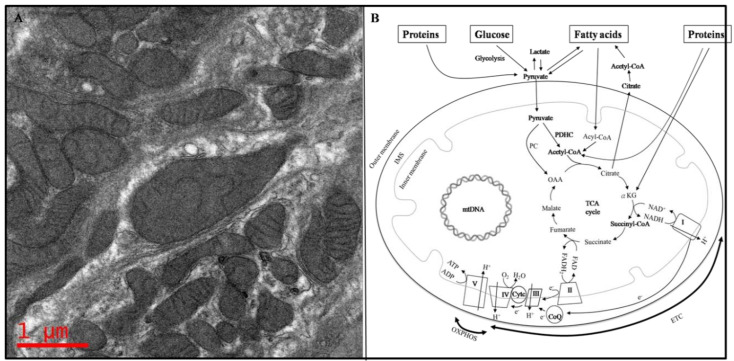
Structure and metabolic pathways in mitochondria. (**A**) Ultra-structure of normal mitochondria (original magnification 34,000×). (**B**) Schematic representation of oxidative phosphorylation. The TCA cycle, which is a series of chemical reactions to release stored energy through the oxidation of acetyl-CoA derived from carbohydrates, fats, and proteins, produces the reducing equivalents NADH and FADH2, which can transport e^−^ to the mitochondrial respiratory chain (or electron transport chain, ETC). When e^−^ are passing through the complexes in the inner membrane of mitochondria, a mitochondrial membrane potential is formed and generates ATP. These reactions, known as oxidative phosphorylation (OXPHOS), require oxygen. The mitochondrial ETC contain five enzymatic complexes (I–V), ubiquinone (or coenzyme Q10, CoQ), and cytochrome c (Cytc).Complexes I, III, and IV pump protons out from the mitochondrial matrix to IMS. Complex II connects the TCA cycle to ETC. Complex IV (cytochromec oxidase; COX) receives an electron from each of four cytochrome c molecules and transfers these electrons to one dioxygen molecule, converting the molecular oxygen into two molecules of water. During this process, Complex IV binds four protons from the inner aqueous phase to form two water molecules and translocates four additional protons across the membrane, increasing the difference in the transmembrane electrochemical potential. Complex V synthesizes ATP from ADP and phosphates utilizing the energy provided by the proton electrochemical gradient. ADP: adenosine diphosphate; ATP:adenosine triphosphate; CoQ:coenzyme Q; Cytc:cytochromec; e^−^: electrons; FAD:flavin adenine dinucleotide; *α*-KG:*α*-ketoglutarate; mtDNA: mitochondrial deoxyribonucleic acid; NADH:nicotinamide adenine dinucleotide; OAA:oxaloacetate; PC:pyruvate carboxylase; PDHC: pyruvate dehydrogenase complex; TCA: tricarboxylic acid.

Mitochondria are essential to life, and up to 1000 mitochondria are present per cell [1]. Although a report showed that mitochondria are approximately 0.75–3 µm^2^ in size [2], in fact, the length, diameter, and number of mitochondria vary widely according to cell type, physiological status, and pathological conditions [3]. Structurally, mitochondria are rod-shaped organelles that appear in various forms, ranging from numerous small individual organelles, as typically depicted in textbook illustrations, to a single large interconnected and membrane-bound tubular network, depending on environmental conditions, cell type, and organism [4]. Mitochondria are surrounded by outer and inner membranes (Figure 1A). There are two distinct regions in the inner membrane: the inner boundary membrane (IBM) and the cristae membrane (CM). The IBM is adjacent to the outer membrane, whereas the CM is the protruding part of the IBM that invaginates into the matrix space, which encompasses diverse enzymes, ribosomes, transfer RNAs (tRNAs), and mitochondrial DNA (mtDNA) (Figure 1B) [5].

### 1.2. Mitochondria, the Powerhouse of the Cell

The functions of mitochondria include the biosynthesis of amino acids, fatty acids, vitamin cofactors and iron-sulfur clusters [6], as well as cell signaling [7] and apoptosis [8]. Energy production is the most crucial function of the mitochondria [9]. No cellscan remain alive without adequate energy supplements. Energy generated in mitochondria is derived from the metabolism of glucose, fatty acids, and proteins to form acetyl-coenzyme A (acetyl-CoA) that enters the tricarboxylic acid (TCA) cycle to form intermediates that carry out the cycle. This cycle, which occurs in the matrix of the mitochondria, provides substrates for the electron transport chain complexes (ETC) which ultimately generates ATP. Therefore, TCA is the final common pathway for the metabolism of these nutrients (Figure 1B).

Glucose metabolism pathway: When tissues or cells uptake glucose, glucose is broken down into two pyruvate molecules through glycolysis. When pyruvate is formed in the cytosol, it is then reduced to lactate, transaminated to alanine, or transported into the mitochondria where it is oxidatively decarboxylated to acetyl-coenzyme A (acetyl-CoA) through the pyruvate dehydrogenase complex (PDHC) and enters the TCA cycle. Additionally, pyruvate can form oxaloacetate (OAA) through pyruvate carboxylase (PC) entering the TCA cycle [10].

Protein metabolism pathway: Proteins are digested by proteases to generate amino acids. Amino acid derivatives, for example alpha-ketoglutarate (derived from glutamate or glutamine), can enter the TCA cycle as intermediates; some amino acids, such as leucine, isoleucine, lysine, phenylalanine, tryptophan, and tyrosine are converted into acetyl-CoA to enter the TCA cycle. In the case of alanine, cysteine, glycine, serine, and threonine, they are metabolized to pyruvate, which converts into OAA or into acetyl-CoA to enter the TCA cycle [11].

Fatty acid metabolism pathway: Fatty acids are transported across the inner mitochondrial membrane to form fatty acyl-CoA, which then forms acetyl-CoA through oxidation in the cytosol and enters the TCA cycle to release ATP from the ETC, which accepts energy-rich hydrogen atoms from nicotinamide adenine dinucleotide (NADH) or flavineadenine dinucleotide (FADH), produced mainly in the TCA cycle and from fatty acid oxidation. Energy is generated in the process as electrons (e^−^) from hydrogen are transported between the ETC. The mitochondrial ETC are known as the “powerhouse of the cell”, where energy generation occurs via oxidative phosphorylation [12]. The mitochondrial ETC, located in the inner membrane of the mitochondria, contain five enzymatic complexes (I–V), ubiquinone (or coenzyme Q10, CoQ), and cytochrome c (Cytc). Complexes I, III, and IV pump protons out from the mitochondrial matrix to IMS. Complex IV receives an electron from each of four cytochrome c molecules, and transfers these electrons to one dioxygen molecule, converting the molecular oxygen into two molecules of water. During this process, Complex IV binds four protons from the inner aqueous phase to form two water molecules and translocates four additional protons across the membrane, increasing the difference in the transmembrane electrochemical potential. Complex V synthesizes ATP from ADP and phosphates utilizing the energy provided by the proton electrochemical gradient [13]. Eventually, all the energy is accumulated in the form of ATP.

### 1.3. Mitochondrial Genetics

Mitochondria possess their own replicating genetic system, and each one contains 100 to 10,000 copies of mitochondrial DNA (mtDNA) according to cell type and developmental stage [1]. Human mtDNA is an approximately 16-kilobase circular double-stranded DNA. This multi-copy genome contains 37 genes, including 13 that encode for subunits of ETC: seven for complex I, one for complex III, three for complex IV, two for complex V (ATP synthase), 22 for mitochondrial tRNAs and two for rRNAs (12S and 16S). Complex II, the remaining subunits of ETC, and the factors associated with mtDNA replication, transcription, and translation are encoded in nuclear DNA [14]. This dual genetic control of the ETC is unique to mitochondria. mtDNA, ismaternally inherited in mammals; therefore, mitochondrial genetics do not obey Mendel’s laws of genetic inheritance. Clinical manifestations of mitochondrial disorders (MD) may be due to mtDNA mutations, including point mutations or complex rearrangements of mtDNA as well as nuclear mutations, leading to mitochondrial DNA depletion or deletions [15]. Heteroplasmy refers to cells and mitochondria containing two populations of mtDNA, a normal and a mutated one. Homoplasmy means that all mtDNA copies in a eukaryotic cell are identical. Thus, levels of mutated mtDNA can be significantly variable in different individuals and tissues according to the extent of the oxidative metabolism they rely on [16]. Clinical manifestations of mitochondrial disorders may be present if the percentage of mutated mtDNA in a cell or tissue surpasses the threshold each tissue has (threshold effect) [16,17]. Additionally, mitotic segregation of mtDNA, whether mutated or normal, may influence the functions of mitochondria. Nuclear factors determining mtDNA segregation in different tissues have been reported [18]. Thus, mutation load, threshold effect and mitotic segregation may explain the different phenotypes in the MD.

## 2. Clinical Manifestations of Mitochondrial Encephalomyopathy, Lactic Acidosis, and Stroke-Like Episodes (MELAS) Syndrome

**Figure 2:**
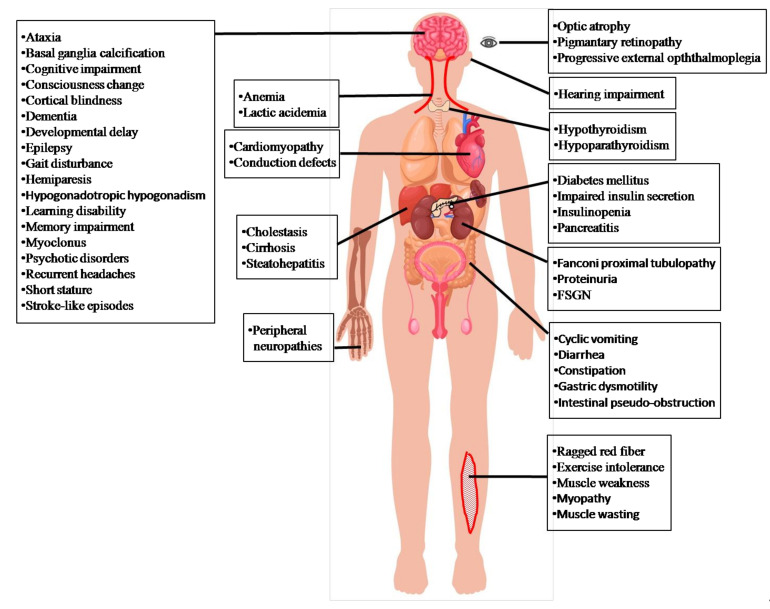
MELAS syndrome manifestations. The clinical features of MD are not specific and are variable between patients, including neurological and non-neurological presentations. MELAS, a common MD, is a progressive syndrome where patients can recover from one phenotype and develop others later. Subjects with mtDNA mutations can be asymptomatic or have multi-organ involvement.

The prevalence of MD is at least 1:8500 of all live births [19]. Among MD, MELAS syndrome is common and well-known in mitochondrial encephalomyopathies. The prevalence of MELAS syndrome has been estimated to be 0.18:100,000 in Japan [20], 1.41:100,000 in the north east of England [21], 2:100,000 in Sweden [22], 18.4:100,000 in Finland [23], and 236:100,000 in Australia [24]. Statistical data on the onset ages of MELAS syndrome showed that 65–76% starts before the age of twenty, 5–8% before the age of two, and 1–6% after forty years old [20,25,26], suggesting that clinical presentations of MELAS syndrome are more common in children than in adults. MELAS syndrome affects various organs, including neurological (the central and peripheral nervous, psychiatric, ophthalmologic, otological) and non-neurological (cardiac, digestive, endocrine, renal, hematological, and muscle) systems [27]. Lungs, stomach, and skin are less frequently affected. Brain and muscle are always seriously damaged by mitochondrial dysfunction [28] (Figure 2).

**Figure 3:**
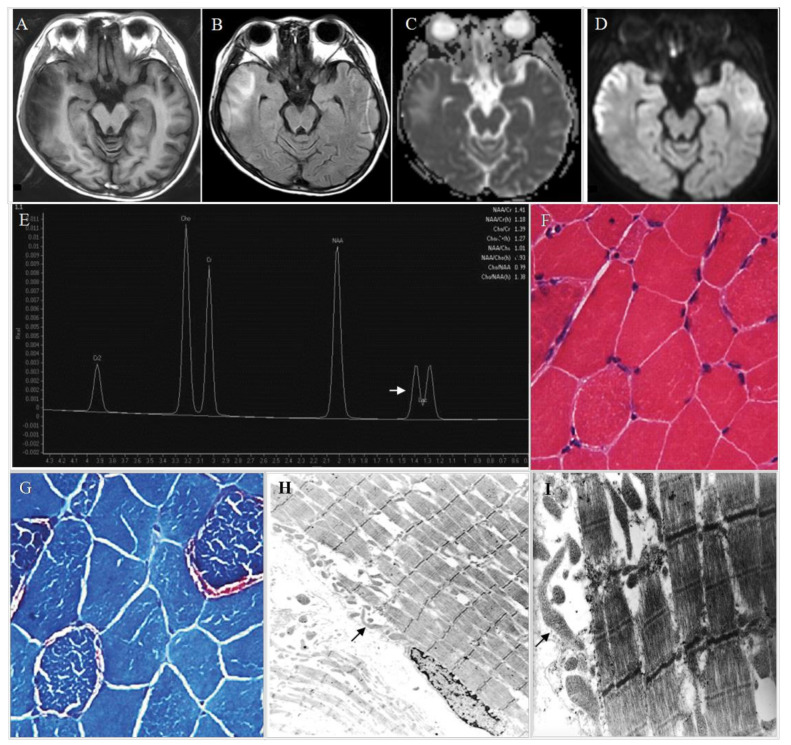
Characteristic findings of MELAS. (**A**) Axial T1-weighted imaging shows focal hypointensity involving the right temporal lobe cortex and subcortical white matter; Gyral swelling is noted. (**B**) Axial FLAIR imaging reveals focal hyperintensity in the same area of the right temporal lobe, and abnormal thickening of the cerebral cortex. (**C**,**D**) Diffusion weighted imaging (DWI) shows restricted diffusion as bright signal intensity along the right temporal lobe cortex; the corresponding area appears as dark signal intensity on the ADC map, compatible with an infarction area. The findings that the area of restricted diffusion in DWI commonly appears with a high signal on the ADC map may be used to distinguish stroke-like episodes from hemodynamic infarctions.(**E**) Proton MR spectroscopy localized to the right temporal lobe of the same patient confirms elevation of lactate doublet at 1.3 ppm (arrow). (**F**) Hematoxylin and eosin staining of muscle histology show focal scattered fibers with clear rim (200×).(**G**) Gomori trichrome staining of ragged red fibers (200×). (**H**) Electron micrographs show focal disruption of myofilaments with accumulated elongated, bizarrely-shaped mitochondria(arrow) in the subsarcolemmal and in the interfibrillar space (3000×). (**I**) Disruption of myofilaments and bizarrely-shaped mitochondria (12,000×).

Central nervous system: Stroke-like episodes are the most typical feature of clinical manifestations of MELAS. Other manifestations include headaches, altered mental status, seizures, partially reversible aphasia, cortical vision loss, and motor weakness. Seizure-associated MELAS syndrome may possibly activate one or more stroke-like episodes [29], which are suggested to be mediated by ictal activity [30]. Severe mitochondrial complex I defects and the preferential loss of inhibitory inter-neurons can potentially lead to neuronal hyper-excitability [31].These clinical manifestations may progressively develop and eventually cause neurological deficits [20,25,26]. Brain images show as nearly normal or appear with stroke-like episodes, cortical atrophy, white matter lesions (Figure 3A–D), and corpus callosum hypo- or agenesis [25,26]. MR spectroscopy demonstrates reduced N-acetylaspartate signals and increased lactate peaks (Figure 3E) [26]. Affected regions may present asymmetric infarction, mainly the temporal, parietal, and occipital lobes (Figure 3A–D). The damage may be restricted to cortical or subcortical white matter. Dementia appears in 40–90% of MELAS syndrome cases [20,25,26], and epilepsy, in which generalized or focal seizures occur, is present in 71–96% [20,25,26]. Repeated stroke-like episodes may increase neurological morbidities and progressive mental deterioration, leading to a poor prognosis [20,25,26]. Other neurological manifestations are recurrent headaches [20,25,26], developmental delay, learning disorders, memory loss, myoclonus, ataxia, altered consciousness, basal ganglia calcifications in neuroimaging, elevated protein in the cerebrospinal fluid (CSF) [25], motor or speech delay, small head circumference, and lower Karnofsky score at baseline [32].

Peripheral nervous system: Axonal or mixed axonal and demyelinating neuropathy in the electrophysiological studies [26,33,34].

Psychiatric: Anxiety, bipolar disorder, depression, psychosis, and personality changes [35].

Ophthalmologic: Ophthalmoplegia, optic atrophy, and pigmentary retinopathy [25].

Otologic: Patients with MELAS syndrome may have hearing problems [20,25,26], including early-onset, mild and progressive sensorineural hearing loss as well as peripheral neuropathy associated with chronic and progressive hearing loss [26].

Cardiac: Patients with MELAS syndrome present symptoms of cardiomyopathy such as dilated and hypertrophic heart [20,25,26], and cardiac conduction defects such as Wolff–Parkinson–White syndrome [25,36].

Digestive: Individuals with MELAS syndrome can present gastrointestinal symptoms such as constipation, diarrhea, gastric dysmotility, intestinal pseudo-obstruction, recurrent or cyclic vomiting and recurrent pancreatitis [20,25,26,37].

Endocrine: Diabetes, type 1 or type 2, is present in 21–33% of MELAS cases [20,26], caused by insulin deficiency, increased gluconeogenesis, and insulin resistance [38]. Mitochondria with mutation-associated energy deficiency cause insulin secretion impairment and insulinopenia [26,39].Nitric oxide (NO) impairment hinders vasodilation, altering the metabolic pathway of glucose and insulin to muscle tissue and thus contributing to insulin resistance [40,41]. Short stature in individuals with MELAS syndrome may be due to chronic energy deficiency [20,25,26]. Growth hormone deficiency is occasionally present, leading to growth retardation [42]. Hypothyroidism, hypogonadotropic hypogonadism, and hypoparathyroidism have been reported in patients with MELAS syndrome [43,44,45].

Renal: Renal manifestations include proteinuria, focal segmental glomerulosclerosis, andFanconi tubulopathy [46].

Blood: Anemia [47].

Muscle: myopathy due to the results of mutations in mitochondria initially manifests as exercise intolerance and proximal muscle weakness [20,25,26].The mtDNA A3243G heteroplasmy in muscle is reported to be associated with maximum oxygen uptake and workload, resting plasma lactate levels, and muscle morphology abnormalities [48].

NO deficiency causes exercise intolerance and attenuates basal muscular perfusion through weakening exercise-induced hyperemia; NO deficiency contributes to muscle wasting and myopathy through reduced synthesis of muscle proteins [40].

## 3. Genetics and Pathogenesis of MELAS Syndrome

### 3.1. Genetics of MELAS Syndrome

At least 1500 mitochondrial genes are predicted to cause mitochondrial dysfunction if they are mutated [49], and at least 30 different gene mutations are identified in MELAS syndrome (MITOMAP, at www.Mitomap.org) [50]. Even though A3243G of mtDNA is the most studied, the mechanisms underlying the mtDNA A3243G-related clinical heterogeneities are still vague. The finding of an adenine to guanine transition at position 3243 (A3243G) of the mtDNA was the starting point for exploring the molecular basis of MELAS syndrome. The missense mutation maps to a conserved residue in the MT-TL1 gene coding for tRNA-leu(UUR), which is presumably used to impair the synthesis of mitochondrial protein [51].Apart from MELAS syndrome, the phenotypes associated to the mtDNA A3243Gmutation are variable between patients, including neurological and non-neurological presentations which range from asymptomatic carriers to severe phenotypes; mtDNA A3243G accounts for approximately 80% of the causative mutations. [26,52]. Other mitochondrial syndromes including Myoclonic Epilepsy with Ragged Red Fibers syndrome and Leigh syndrome are reported to be associated with this mutation [53,54]. Several other mtDNA mutations, such as T3271C, A 3252G, T3291C, G3959A, A10134C, T10191C, G10197A [55], G13513A [56], and T10158C [57] as well as mutations in nuclear genes such as the polymerase gamma 1 (POLG1) [58] and PDHC deficiency [59] can also cause MELAS syndrome.

### 3.2. Pathogenesis of Stroke-Like Episodes of MELAS Syndrome

The pathogenesis of the stroke-like episodes in MELAS syndrome remains controversial. At least three mechanisms, including insufficient energy, angiopathy with impaired blood perfusion, and NO deficiency have been proposed [26,40,60,61,62].

#### 3.2.1. Insufficient Energy

It is proposed that the dysfunctional mitochondria may not be able to produce sufficient energy to support various vital organs, leading to multi-organ impairment [63]. Brain MRI spectroscopy shows increased lactate peaksand decreased NAApeaks over the occipital region during an acute episode; levels of those metabolites may return to normal after clinical resolution [64,65], suggesting that one of the underlying mechanisms of the stroke-like episodes of MELAS is ischemic insult, and could possibly be reversible. At the molecular level, lactic acidosis in MELAS syndrome can be attributed to a substantial reduction and dysfunction in mitochondrial ETC subunits and OXPHOS activity, leading to insufficient ATP production and overproduction of lactate [19,66].Therefore, this scenario points to energy failure as a root cause, perhaps exacerbated by increased energy demand during concurrent infections or seizures, eventually leading to neuronal or multi-organ damage.

#### 3.2.2. Angiopathy

Brain MRIs of patients with MELAS syndrome typically show multifocal, symmetric, infarct-like lesions. These lesions are mainly located in the temporal and occipital lobes, instead of the entire cerebrum, and extensive neuronal loss is commonly noted in the cerebral cortex and the cerebellum [67]. However, these lesions are inconsistent with the arterial blood supply [68]. Muscle biopsies from MELAS patients demonstrate proliferated mitochondria aggregated in the endothelial cells and smooth muscle of small vessels in the cerebral and cerebellar areas [67].At the cellular level, abnormal mitochondria are found in the endothelial cells and smooth muscle of pial arterioles, on the surface of the brain, and in the Virchow–Robin space as well as in the small intracerebral arteries [69].These mitochondria exhibit abnormal sizes and shapes as well as inclusions in the capillary walls of the brain of patients with MELAS syndrome [70,71], and cause micro- or macro-angiopathy, leading to narrowing of the vessel lumen and thus impeding blood flow and impairing perfusion. These findings led to the term “mitochondrial angiopathy” [71]. Apart from reversible vasoconstriction syndrome causing cerebral ischemia, the brain has relatively feeble protective mechanisms against the oxidative stress caused by these abnormal mitochondria generating excessive reactive oxygen species (ROS) [72];these directly or indirectly damage neurons and endothelial cells, leading to the clinical manifestations of MELAS syndrome. Consistently, PET imaging shows increased oxidative stress and glucose metabolism following hyperemia in a patient with MELAS syndrome and stroke-like episodes [70]. However, the insufficient energy and mitochondrial angiopathy cannot explain how the mtDNA A3243Gmutation, which exists all over the brain, causes focal brain lesions.

#### 3.2.3. NO Production Deficiency

**Figure 4:**
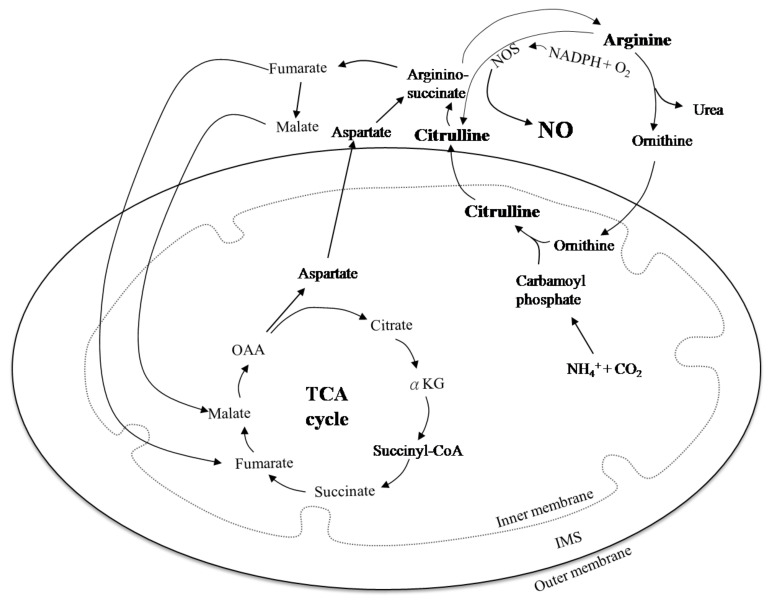
Schematic representation of arginine metabolism. Carbamoyl phosphate interacts with ornithine and releases a phosphate group converted to citrulline through ornithine transcarbamoylase. The TCA cycle begins with condensation of acetyl-CoA and oxaloacetate (OAA) to produce citrate. Aspartate and citrulline form argininosuccinate via argininosuccinate synthetase. Argininosuccinate is cleaved by argininosuccinase to generate fumarate and arginine. Fumarate produced in the cytosol can translocate into the mitochondria, where it can serve as a substrate for the mitochondrial fumarase, which catalyzes its hydration into malate. Arginine undergoes cleavage by arginase to produce ornithine and urea. Ornithine is shuttled back to the mitochondria to roll the urea cycle. Nitric oxide synthases (NOSs) hydroxylate arginine to generate *N*-hydroxy-l-arginine (NOHA), which is oxidized by the enzyme to generate citrulline and NO, with NADPH and O_2_ serving as co-substrates.

The activity of cytochrome c oxidase (COX; complex IV) is presumed to be linked to the mtDNA A3243G mutation [73], and therefore mtDNA A3243G mutation may affect the functions of COX. However, the hippocampus, which harbors the highest percentage of COX-deficient neurons, only exhibits minor neuronal loss; the occipital lobes, without COX-deficient neurons, manifest the most severely damaged areas [70]. COX knockout mice show cardioencephalomyopathy with a concomitant decrease of mitochondrial copper content [74], which does not appear in MELAS syndrome. Also, most MELAS mutations result in deficient complex I instead of complex IV [75]. Consistent with this, an autopsy report showed anA3243G MELAS patient with a significant reduction in complex I activity and a slight reduction of complex IV in her brain [76], suggesting that the mutations of COX are irrelevant to the phenotypes of A3243G MELAS. Even so, cases of COX-negative fibers in patients with MELAS syndrome are still reported [77]. Interestingly, this study discovered a reduction of n-nitric oxide synthase (n-NOS) activity in these COX-negative fibers. NOS has three isoforms: neuronal NOS (nNOS; NOS-1), inducible NOS (iNOS; NOS-2), and endothelial NOS (eNOS; NOS-3) [78]. NOS converts arginine to citrulline and also generates NO (Figure 4). NO is a pivotal signaling molecule that regulates blood flow and tissue oxygenation [79], as well as relaxing vessels to achieve flow-mediated vasodilation [80]. However, flow-mediated vasodilation is impaired in patients with MELAS [81]. Low levels of NO have been reported in MD, including MELAS [82]; possible causes include dysfunctional endothelial cells leading to the reduction of NO synthesis, lower concentrations of arginine and citrulline (which are NO precursors), higher concentrations of asymmetric dimethylarginine (which is an NOS inhibitor), and NO scavenging [82].

MELAS patients were reported to show lower NO metabolite levels, including L-arginine [38,83] and L-citrulline [61,83] during stroke-like attacks. Therefore, NO depletion may play a significant role in the pathogenesis of several MELAS syndrome-associated phenotypes [84]. Citrulline can be metabolized to arginine through argininosuccinate lyase and argininosuccinate synthase (Figure 4); accordingly, both arginine and citrulline may act as NO donors [85] and, apparently, citrulline may, like arginine, potentially provide therapeutic effects in MELAS patients.

## 4. Diagnosis

**Figure 5:**
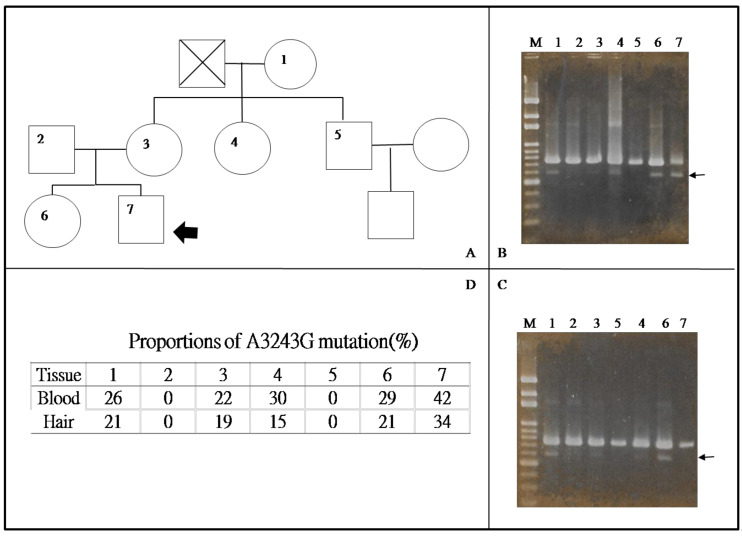
A patient with MELAS syndrome with his family members carrying the heteroplasmic mtDNA A3243G mutation. (**A**) The pedigree of the family. Arrow indicates the proband, who had typical features of MELAS syndrome including seizures, lactic acidemia, headache, hemiparesis, hemianopsia, stroke-like episodes, hearing impairment, and mental deficits. His family members (3, 7) are asymptomatic. Levels of mutant mtDNA in the (**B**) blood and (**C**) hair follicle. (**D**) Quantification the ratio of mutation mtDNA A3243G of the B and C. M: 100–1000 bp DNA marker. Our results show that ratio of mutation is higher in the proband than in his family, and the ratio of mutation is higher in subjects with symptomatic presentations than asymptomatic carriers.

Pavlakiset al. first proposed the diagnostic criteria for MELAS syndrome, including the onset of symptoms between the ages of 3 and 11, normal early development, short stature, seizure and alternating hemiparesis, hemianopia (or cortical blindness), ragged red fibers (RRF, Figure 3G), lactic acidemia, and parieto-occipital lucencies in brain computed tomography scans [86]. However, the phenotypes of MELAS are extremely variable, and clinical features of MELAS syndrome are not specific and may also be present in other MD [87]. A muscle biopsy with appropriate staining may provide useful information [88]. Furthermore, ultrastructural investigation can demonstrate unique pathologies in MELAS patients, including mitochondrial accumulation among muscle fibrils and more prominently in the subsarcolemmal region, as well as enlarged, elongated, ring- or bizarrely-shaped mitochondria(Figure 3H,I). Cristae in such mitochondria may be concentric or thickened, and paracrystalline inclusions may be observed. However, these findings can be detected almost in other types of mitochondrial myopathies [89]. Due to the lack of specificity, mitochondrial alterations with electron microscope evaluation have low priority in the diagnostic procedure of MELAS syndrome. In fact, the key clues to the diagnosis of MELAS are the manifestations of a stroke-like episode and encephalopathy with dementia and/or seizures at a young age [90,91]. The MELAS Study Group in Japan has developed their diagnostic criteria based on Hirano [90] and Hirano and Pavlakis [25], including two categories. Category A consists of clinical presentations of stroke-like episodes, while category B consists of evidence of mitochondrial dysfunction. A definitive diagnosis of MELAS syndrome should include two items in category A and two items in category B (four items or more), while a diagnosis of supportive MELAS syndrome should include one item in category A and two items in category B, and at least three items. Moreover, the Group emphasizes the importance of detecting the genes involved in both mtDNA and nuclear DNA that connect the phenotype in making the diagnosis of MELAS syndrome. Essentially, it is crucial to quantify the ratio of mtDNA harboring wild-type and pathogenic mutations so as to understand the disease progression of MD and to evaluate the effects of therapeutic approaches. Leukocytes, hair follicles, urinary sediment, buccal mucosa, saliva and skeletal muscle tissue can all be used for diagnostic testing [92,93]. Although the mtDNA A3243G mutation can be detected in blood leukocytes, the level of mutation declines over time [94], resulting in very low or undetectable levels inpatients severely affected with MELAS syndrome [95]. Studies have shown that the analysis of blood samples is not helpful for predicting the prognosis of a patient with MELAS syndrome, as no obvious correlations exist between the mutant load in blood and a patient’s clinical features [95,96]. Moreover, the mtDNA A3243G mutation load in the blood is likely a poor indicator of the overall mutation load in affected tissues, and is therefore not a good candidate for noninvasive diagnostic testing [97]. For instance, the proportion of mutant mtDNA in blood or hair follicle samples is higher in patients with MELAS syndrome than in their family members with few or no symptoms (Figure 5, subject 7). As shown in Figure 5, the proportion of mutant mtDNA in the blood and hair follicle samples of the proband was somewhat low. Numerous reports have suggested that urine sediment may represent a better test material than blood because the mtDNA A3243G mutation level in urine is consistently higher than that in blood and typically reflects the mutation load present in skeletal muscle [97,98,99]. This is likely due to the presence of urinary epithelia, which derive from the endodermal germ layer. Each germ layer gives rise to a tissue that can demonstrate high levels of this mutation, which indicates that the initial mutation level is equal in all layers throughout the embryo [100]. Therefore, urine has become a useful sample for assessing disease severity [97,98,99].

A cardinal sign of MELAS syndrome is lactate acidosis. Patients with MELAS syndrome may show elevated lactic acid and pyruvic acid levels in plasma and cerebrospinal fluid (CSF) [71,86,101], and evidence suggests that high lactate levels are associated with increased mortality [32]. Although the affected regions identified by neuroimaging do not correspond to the classical vascular distribution pattern, the lactate levels measured by MR spectroscopy correlate with the degree of neurological impairment and the short survival of patients with MELAS syndrome [102], supporting previous observations that elevated lactate levels, especially in the CSF, may be associated with increased disease severity [32,65]. Although the literature reports a strong link between the severity of the phenotype and the degree of the mutation load [103], postmortem studies have not supported this correlation [75,104,105]. Moreover, patients with similar heteroplasmy levels present different symptoms; patients with high heteroplasmy levels show few or no symptoms [106]. A report showed that individuals with >75% heteroplasmy of mtDNA A3243G mutation did not harbor MELAS syndrome phenotypes [17], suggesting that high levels of the mtDNA A3243G in tissues may not truly express the phenotypes. Factors such as mtDNA copy number and nuclear factors may be involved in the presence of these phenotypes. Advanced molecular diagnostic tools, such as whole-exome sequencing [107] or next-generation sequencing [108], can help identify nuclear and mitochondrial mutations. Due to the specificity and convenience of these noninvasive genetic tests, very few patients with MELAS undergo biopsies. Muscle biopsy alone can provide some information in patients with suspected MELAS syndrome, but the diagnosis is not confirmed by genetic testing [109].

Pre-implantation genetic diagnosis (PGD) is a reproductive strategy for mtDNA mutation carriers which serves as a preventive method for reducing the likelihood that patients with known mtDNA mutations will pass them to their offspring [110]. The procedure involves the in vitro fertilization of oocytes harboring pathogenic mtDNA mutations which are cultured to the 6–8 cell stage, at which time one or two cells are sampled for mutational load analysis [111] or cultured for five days and biopsied at the blastocyst stage [112]. Cells from either stage without the mutation or with mutational loads below the phenotypic threshold are then transferred to the uterus. PGD allows specialists to assess the level of mutated mtDNA likely to be passed from mother to child. However, PGD has several limitations for. First, PGD is not suitable for women with homoplasmic mtDNA mutations [113]. Second, while the mutational load determined at the time of embryo biopsy is thought to be representative of the entire embryo, the timing of the test is crucial to the results. Third, the mutational load presumed to remain constant during fetal development has not been verified [114]. Fourth, PGD may fail to identify an embryo with a low risk of mtDNA disease, resulting in selection for transfer. Furthermore, the threshold effect can vary for different disease types and mutations [115].

### 4.1. Treatment

Patients with MELAS syndrome and their families benefit greatly from a multidisciplinary approach to care, especially social workers and physical therapists as these professionals can help improve quality of life for these patients [88]. Management of this disease is mainly symptomatic. Supportive treatment includes adequate fluid, nutrition, and medication and anti-psychotic or sedative therapy, as well as rehabilitation. Seizures commonly occur in patients with MELAS syndrome [116] because lesions that develop during stroke-like episodes lower seizure thresholds, resulting in a predilection for prolonged focal seizures [117,118]. Epileptic seizures in patients with MELAS syndrome should be aggressively treated because neurons utilize glycogen as an ATP source during epileptic activity [119]. Glycolytic by-products such as methylglyoxal can accumulate and become toxic to neurons and astrocytes at higher concentrations, leading to neuronal impairment, breakdown of the blood–brain barrier (BBB), and vasogenic edema, triggering further seizure activity [119]. Recurrent, unexpected acute lesions are induced by stroke-like episodes, leading to neuronal hyperexcitability, focal hyperemia, inflammation, necrosis, and edema [118,120,121]. Additionally, prolonged seizures may lead to neuronal injury and a distorted BBB [119]. These events combine to make seizures in MELAS patients extremely difficult to control. The choice of antiepileptic drugs is complicated by the high potential for mitochondrial toxicity associated with valproic acid, carbamazepine, phenytoin, and phenobarbital [29].Metformin is the first-line drug for the treatment of type 2 diabetes. However, metformin is contraindicated in patients with MD, especially MELAS and diabetes, due to the predisposition for lactic acidosis [122].

Prolonged seizures in patients with MELAS syndrome have been proposed to damage the BBB [119], and the use of antiepileptic drugs can minimize the occurrence of stroke-like episodes [119]. Although the mechanisms of action through which steroids exert their effects during the treatment of MELAS syndrome remain largely unknown, steroid responsiveness is consistently observed in MELAS patients, and steroid withdrawal results in deterioration of the condition [67]. Several case reports have shown that steroids are effective for preventing the progressive spread of stroke-like episodes in patients with MELAS syndrome [123,124], and a cohort of patients with mitochondrial leukoencephalopathy showed partial or complete steroid responsiveness [125]. Steroids may help to stabilize the BBB during the acute stage of a stroke-like episode [119] and promote the functional recovery of the BBB after blast injury [126].

Dietary supplements are month combined regimen including 25 mcg/day of biotis (CoQ_10_, vitamin C, folic acid, vitamin E, N-acetylcysteine), agents related to modulating ETC(vitamin B1, B2, B3, folic acid andCoQ_10_), NO precursors (Levoarginine), energy buffers (creatine), and agents related to metabolism (Biotin, B12, Levocarnitine) and mitochondrial biogenesis (vitamin B3). In addition to dietary supplements, a proper aerobic training program can improve the neuromuscular functions of patients with MELAS syndrome [127,128]. Moreover, a new technique, known as mitochondrial replacement therapy (MRT) can replace defective mitochondria with healthy varieties obtained from a female donor before or after an egg is fertilized, providing women with MD with a chance to have unaffected children [129].

#### 4.1.1. Vitamin B1

The Vitamin B1 (thiamine)recommended dose for children < 3 years is 150 mg/day, for children > 3 years, 300 mg/day, and for adults, 900 mg/day, PO [130]. Mammalian cells cannot synthesize thiamine endogenously but need to obtain it from the surrounding environment and convert it to thiamin pyrophosphate (TPP) in the cytoplasm. Thiamine provides an important link between the glycolytic and TCA cycles because it plays an important role in the pentose phosphate pathway, which is not only an alternate glucose metabolism pathway but also a pathway for the synthesis of numerous neurotransmitters, nucleic acids, lipids, amino acids, and steroids. Thiamine is also involved in the TCA cycle, including decarboxylation of pyruvate and oxidation of α-ketoglutamic acid; Thiamine is a cofactor of α-ketoacid dehydrogenases, which are involved in oxidative energy metabolism, ATP synthesis, and reduction of cellular oxidative injury [131]. Therefore, vitamin B1 serves a critical role in the functional and structural homeostasis of the mitochondria [132].

#### 4.1.2. Vitamin B2

The Vitamin B2 (riboflavin) recommended dose is 50–400 mg/day, PO [130]. Vitamin B2 is a water-dissolved nutrient with multiple functions including nourishing skin and hair, anti-oxidation, and regulating the immune system. Vitamin B2 and its derivativesflavin adenine dinucleotide (FAD) and flavin mononucleotide (FMN) play crucial roles as cofactors for enzyme-catalyzed reactions related to the respiratory chain [133]. Riboflavin is necessary for normal development, lactation, physical performance, and reproduction [134]. As an increased dose of vitamin B2 and its derivatives can theoretically propel cellular oxidative phosphorylation to generate more ATP, a case report showed that a riboflavin dose of 300 mg/day plus 4 g/day nicotinamide for 18 months improved symptoms of encephalopathy in patients with MELAS syndrome [135]. Vitamin B2 may have beneficial effects against MELAS syndrome because vitamin B2 and CoQ_10_ are reported to restore the pathological changes in patient-derived fibroblasts and cybrid models of MELAS syndrome [136].

#### 4.1.3. Vitamin B3

The Vitamin B3 (niacin) recommended dose is 10 mg/kg/day, PO [130]. Nicotinamide, a precursor of nicotinamide adenine dinucleotide (NADH), which is the primary e^−^ donor for respiratory chain complex I, is involved in many biological reactions. The use of vitamin B3 supplementation is reported to improve cellular NAD^+^/NADH balance and enhance mitochondrial biogenesis in human fibroblasts with complex I dysfunction [137]. Vitamin B3 supplementation is also reported to improve the survival of a complex I disease in the C. elegans model [138].

#### 4.1.4. Vitamin B7

The Vitamin B7 (biotin)recommended doses is 2–10 mg/day, PO [130]. Biotin, a water-dissolved nutrient, acts as an essential coenzyme for five carboxylases involved in the digestion of carbohydrates, synthesis of fatty acids, and gluconeogenesis [139]. An open-label trial investigated the effects of a 12-month combined regimen including 25 mcg/day of biotin,CoQ_10_, carnitine, vitamin C, vitamin K1, riboflavin, thiamine, niacin, pyridoxine, pantothenic acid, cyanocobalamin, and folic acid in twelve MD patients. Only one patient showed improvement in energy metabolism and exercise tolerance, while nine patients showed no effects [140].

#### 4.1.5. Vitamin B9

The Vitamin B9 (folate) recommended dose for folinic acid is 1.5–5 mg/kg/day, PO [130]. Folate exists in several forms, including folic acid, folinic acid (known as 5-formyltetrahydrofolate), L-methylfolate, and 5-methyltetrahydrofolate (5-MTHF) [141]. As functions of folate include DNA and RNA synthesis, creatine synthesis, and homocysteine remethylation [141], folate is essential to human health. However, the human body cannot synthesize folate de novo. Therefore, folate is acquired completely from dietary sources [142]. Folic acid, a water-soluble oxidized synthetic form, does not present in nature [143]. Folinic acid (5-formyltetrahydrofolate), the active form of folic acid, can cross the blood–brain barrier [144]. Grossly, folate deficiency has been reported to increase risk of cancer, cardiovascular disease, cognitive impairment, and neural tube defects [143]. Central folate deficiency, including reduced hippocampal and amygdalar volumes and global brain atrophy [145], are associated with MD [142]. Molecularly, folate deficiency may increase mtDNA deletion and reduce COX gene expression in mitochondria [145], suggesting a link between low folate status and mtDNA instability.

#### 4.1.6. Vitamin B12

The Vitamin B12(B12; cyanocobalamin) recommended dose is 1000 mg/day, PO [146]. The functions of B12 are closely connected with that of folate in cellular metabolism [147]. Either folate or B12 deficiency impairs nuclear de novo synthesis of thymidine and causes instability of the genome [148]. B12 deficiency causes megaloblastic anemia and neurological symptoms such as depression, dementia, and schizophrenia by impairing the synthesis of DNA. Therefore, B12 plays an important role in maintaining hematopoiesis and neurological function [149].

#### 4.1.7. Vitamin E

The Vitamin E recommended dose is 2–10 mg/day, PO [130].Vitamin E functions as a free radical scavenger. While being exposed to polyunsaturated fatty acids (PUFA), the use of vitamin E can attenuate oxidative damage from PUFA by inhibiting the release of inflammatory cytokines and inactivating nuclear factor kappa–light chain enhancer of activated B cells (NFkB). Consequently, vitamin E deficiency is reported to increase the risk of atherosclerosis and other degenerative diseases [150]. Vitamin E and N-acetylcysteine (NAC) are reported to considerably improve lifespan in the genetic-based mitochondrial complex I disease C. elegans model, and to significantly protect against brain death in zebra fish exposed to a toxin with mitochondrial ETC complex I inhibition [151], suggesting that vitamin E may potentially have a protective function in mitochondrial disease.

#### 4.1.8. Coenzyme Q10

The Coenzyme Q10 (CoQ_10_) recommended dose for children is 2–8 mg/kg/day. and for adults, 50–600 mg/day, PO [130]. CoQ_10_, a fat-soluble quinone, is generated in the mitochondrial inner membrane [152]. CoQ_10_ canscavenge dangerous free radicals, block free radical-induced mitophagy [153,154], facilitate e^−^ transfer in the ETC [155], stabilize the ETC, reverse CoQ_10_ deficiencies in MD [136], and enhance ETC activity in patients with impaired complex III function [153]. The effects of CoQ_10_ in MD may reduce serum levels of lactate and pyruvate, improve heart contractile force and conduction defects [156],improve the visual field [157], strengthen muscle weakness [158], enhance oxygen utilization and tolerance in exercise [156], and improve neurological functions [159].CoQ_10_ is recommended for subjects with MELAS syndrome presenting muscle weakness, fatigue, and high levels of lactate [160]. The use of CoQ_10_ is reported to restore the pathological changes in fibroblasts derived from MELAS patients [161]. However, the effects of CoQ_10_ may be restricted to the peripheral nervous system due to the blockade of the blood–brain barrier. Ubiquinol is a reduced form with better bioavailability of CoQ_10_.Idebenone, a synthetic version of CoQ_10_, can cross the blood–brain barrier, has a potent antioxidant activity, and facilitates e^−^ flux along the mitochondrial ETC in order to increase the production of ATP. However, trials have failed to show clinical efficacy, and therefore the Food Drug Administration (FDA) in the United States has not approved it for the treatment of MD [162].

#### 4.1.9. N-acetylcysteine (NAC)

The N-acetylcysteine (NAC) recommended dose is 10 mg/kg/day [130]. NAC can enhance the synthesis of glutathione, which is a main endogenous antioxidant scavenging system in humans [163]. Since levels of glutathione in tissues are commonly low in subjects with MD [164] and NAC is reported to extend life- and health-span in C. elegans, zebrafish, and human cell models of MD [151], NAC may be beneficial to subjects with MELAS.

#### 4.1.10. Vitamin C (Ascorbic Acid)

The Vitamin C (ascorbic acid) recommended dose is 25 mg/kg/day, PO [165]. Vitamin C, also known as ascorbic acid, is essential to growth, development, and the prevention of coronary heart disease, cancer, and stroke [166]. Humans cannot endogenously synthesize vitamin C [167], which is involved in the biosynthesis of collagen, carnitine, catecholamines, and in enzymatic reactions associated with oxytocin, vasopressin, cholecystokinin and alpha-melanotropin [168], as well as in specific plasma membrane transporters [169]. Vitamin C functions as an antioxidant because it can effectively prevent hydroxyl and peroxyl free radicals as well as dioxygen and the superoxide anion [169]. Vitamin C is found to enter mitochondria through Glut1 to protect mitochondria from oxidative stress [170], and is therefore suggested for the treatment of MD [171]. The use of vitamin C in combination with vitamin K is reported to show mild improvement in clinical symptoms in patients with complex III deficiency [171]. However, high doses of vitamin C are toxic in the complex I disease C. elegans model [151].

#### 4.1.11. Levocarnitine (L-carnitine)

The Levocarnitine (L-carnitine) recommended dose for children is 100 mg/kg/day, and for adults 3 g/day, divided into three doses [165]. Carnitine is only active in the levo-isoform; it can elevate the NAD^+^/NADH ratio. Carnitine can translocate protons across the inner membrane of mitochondria to generate ATP (Figure 1B). Levocarnitine can assist long-chain fatty acids transferring to the mitochondrial matrix to drive β-oxidation [165], and can elevate the levels of intracellular acetyl-CoA [153]. Clinical problems are that in patients with mitochondrial cytopathies with complex I dysfunction it may cause a secondary deficiency of long-chain fatty acid oxidation [172]. As one main function of complex I is to initiate the oxidation of NADH, impaired complex I causes the NAD^+^/NADH ratio to fall, diminishing the production of mitochondrial energy [173]. Moreover, low levels of acetyl-CoA impede ATP generation. Therefore, the use of levocarnitine may potentially enhance energy status in subjects with MELAS.

#### 4.1.12. Creatine

The Creatine recommended dose is 5 g/day for adults and 0.1 g/kg day for children [130]. Creatine is endogenously produced in the liver from arginine and glycine and is mainly stored in the muscles, heart, and brain [174]. Creatine also exists in foods such as meat and fish [175]. Creatine in combination with phosphate forms phosphocreatine, which is used as an energy buffer in the mitochondria and as an energy source in tissue during anaerobic metabolism [176]. As the aerobic energy utility in MD patients is dysfunctional and phosphocreatine levels in the skeletal muscle of MD patients are low, exogenous creatine supplementation can enhance the concentration of phosphocreatine in tissues [153,174], suggesting clinical benefit in individuals with MELAS presenting with exercise intolerance and muscle fatigue [165,175]. Combined use of creatine monohydrate, CoQ_10_, and lipoic acid has strengthened muscle power and decreased plasma lactate in subjects with mitochondrial cytopathies, including MELAS syndrome [177]. However, a placebo-controlled crossover trial showed that creatine had no effects on the energy metabolism of skeletal muscle [178].Administering creatine to individuals with A3243G should be used with caution due to the risk of developing focal segmental glomerulosclerosis and renal failure [179].

#### 4.1.13. Levoarginine (L-arginine)

The Levoarginine (L-arginine) recommended dose is 0.5 g/kg for children, or 10 g/m^2^ body surface area for adults [165]. For acute stroke, L-arginine hydrochloride is administered within 30 min of the onset of stroke-like symptoms. If needed, doses may be repeated every 6 h for 1–3 days until the desired effect has been achieved [165]. Studies have shown that an intravenous infusion 0.5 g/kg for seven days during the acute phase improves stroke-like episode symptoms, and oral L-arginine supplementation during the interictal phase decreases the frequency and severity of stroke-like episodes [38,180]. The fact that arginine improves stroke-like episodes in MELAS syndrome is hypothesized to be through increasing NO production, leading to improved intracerebral microcirculation and reduced endothelial dysfunction [61,180]. Additionally, L-citrulline, a precursor of L-arginine, may produce an even larger amount of NO than does L-arginine [61]. L-arginine is involved in urea detoxification, creatine synthesis, and nitric oxide synthesis, as well as in growth and development generally. L-arginine has been proven to show clinical benefits in the treatment and prevention of the stroke-like episodes of MELAS, with long-term data suggesting improved survival and reduced debility of patients with MELAS syndrome [181,182]. Moreover, arginine and citrulline supplementation caused decreased levels of alanine and lactate in plasma, implying that such supplementation may potentially lessen lactic acidemia in MELAS syndrome through enhancing NO production, restoring perfusion, and oxygen delivery [61,183]. Being a substrate of NOS in the vascular endothelium, it is better to intravenously administer L-arginine in order to replenish NO levels and loosen up smooth muscle in the vessels. As chloride overload from intravenous L-arginine may increase the risk of patients developing metabolic acidosis, the administration of L-arginine should be carefully monitored. A study investigating the effects of L-arginine versus placebo for acute stroke-like events in patients with MELAS syndrome showed that all stroke symptoms improved dramatically within 30 min. Levels of L-arginine, pyruvate, NO metabolites, and citrulline returned to normal 24 h after treatment. Regional cerebral blood flow, evaluated with single-photon emission CT scans, showed a focal decrease in the ischemic region 60 min after dosage [38], suggesting that L-arginine therapy improved perfusion in microvasculature compartments and endothelial dysfunction and improved almost all symptoms related to stroke-like episodes, probably through increasing NO availability. A multicenter, prospective clinical trial enrolling 15 and 10 patients with MELAS who received the systematic administration of oral and intravenous L-arginine, respectively, showed that the systematic administration of oral and intravenous L-arginine was therapeutically beneficial and clinically useful for patients with MELAS [184]. However, the drawbacks of this study included a lack of consideration for heteroplasmy rates among mtDNA variants and a failure to consider epileptic activity as a possible driver of stroke-like episodes [185]. In a retrospective study of 71 pediatric patients with MD, 53% of the stroke-like episodes did not respond to L-arginine [181]. A study using patient-derived fibroblasts and cybrid models of MELAS syndrome did not identify any beneficial effects for thiamine, carnitine, creatine, vitamin C, vitamin E, or L-arginine [136], suggesting that the use of L-arginine remains controversial. Therefore, the consensus-based statements for the management of mitochondrial stroke-like episodes in European countries do not recommend the use of this reagent during stroke-like episodes [30].

#### 4.1.14. Aerobic Training

Patients with MELAS syndrome typically present with weakness, fatigue, severe exercise intolerance, and skeletal muscle wasting. However, studies have shown that a well-designed aerobic training program can increase exercise tolerance, enhance the capacity for fractional O_2_ extraction by skeletal muscle, improve the alignment between microvascular O_2_ delivery and O_2_ utilization, increase the efficiency of skeletal muscle oxidative metabolism, improve muscle strength and muscles mass, and increase mitochondrial contents and function [127,128,186,187,188,189,190,191]. These findings indicate that a well-designed aerobic training program can be used as a therapeutic strategy in patients with MELAS syndrome and other MD.

#### 4.1.15. Mitochondrial Replacement Therapy (MRT)

In Mitochondrial replacement therapy (MRT) [192],the nuclear genome is withdrawn from an oocyte or zygotes that harbor mitochondrial mutations and implanted in a normal enucleated donor cell [193]. MRT was originally designed for the treatment of infertility in older women [194]. As most MD have no available treatments, MRT techniques can be used to reconstruct functional oocytes and zygotes to avoid the inheritance of mutated genes and provide women with MD the chance to have unaffected children [129]. However, MRT faces several ethical and theological concerns because a child born using this technique will harbor three distinct genetic materials: one set from the father through the spermatozoa, one set from the biological mother, represented by the nuclear DNA, and a third set from the donor of the cytoplasm containing mitochondrial DNA without pathological mutations, generating a “three-parent baby” [195]. Mismatches between mitochondrial and nuclear genomes may also occur during this process [196].

## 5. Conclusions

MELAS syndrome is a maternally inherited mitochondrial disease with broad manifestations, including encephalomyopathies such as dementia, epilepsy, and myopathy, lactic acidemia, and stroke-like episodes. A multidisciplinary team including a neurologist, an audiologist, a cardiologist, an endocrinologist, a psychologist, an ophthalmologist, rehabilitation therapists, social workers, and genetics professionals is necessary to treat and evaluate patients with MELAS syndrome. Comprehensive neurological examinations, cognitive assessments, brain MRIs, audiology and ophthalmology examinations, growth assessments, echocardiograms, electrocardiograms, and screening for endocrine diseases should be regularly performed in MELAS patients to closely track progression and detect potential complications in the early stages. Genetic counseling should be provided by genetics professionals to any patients and their families in order to provide them with information regarding the disease’s nature, course, and mode(s) of inheritance so as to help proband and family members understand the disease and make well-informed decisions. By acquiring a family history and using genetic testing, a genetics professional may clarify genetic status and analyze the genetic risks among family members. However, because the mutational load detected in the sampled embryonic and fetal tissues may not correspond to the mutational loads in all fetal tissues, and because the mutational load in tissues sampled prenatally may shift in utero or after birth due to random mitotic segregation, the prediction of phenotypes based on prenatal studies cannot be made with certainty, which may puzzle the medical staff, proband, and family members [197]. The mtDNA A3243G mutation is commonly detected in most MELAS patients. Several mechanisms alone or in combination cause the multi-organ manifestations of MELAS syndrome, which include energy insufficiency, angiopathy, and NO production deficiency. Diagnosis of MELAS syndrome is challenging. A careful medical history, physical examination, laboratory tests including blood lactate levels, pH values and genetic studies, brain scans, and most importantly a high level of suspicion, may lead to the diagnosis. Apart from conservative treatment, information on the therapeutic efficacy of dietary supplements used as a supportive treatment for subjects with MELAS syndrome are limited. Only few case reports can be used as references for the therapeutic efficacy of dietary supplements in the patients with MELAS syndrome due to the rarity of the disease. However, most supplements do not have serious adverse effects and some of them may help prevent further deterioration. Certainly, further research is warranted.
